# Mifepristone improves chemo-radiation response in glioblastoma xenografts

**DOI:** 10.1186/1475-2867-13-29

**Published:** 2013-03-25

**Authors:** Monserrat Llaguno-Munive, Luis Alberto Medina, Rafael Jurado, Mario Romero-Piña, Patricia Garcia-Lopez

**Affiliations:** 1 Instituto Nacional de Cancerología, Subdirección de Investigación Básica, Av. San Fernando No. 22, Tlalpan 14000, Apartado Postal, 22026, México, DF, Mexico; 2 Instituto de Física, Universidad Nacional Autónoma de México, México, DF 04510, Mexico; 3 Unidad de Investigación Biomédica en Cáncer INCan-UNAM, Instituto Nacional de Cancerología, México, DF 14080, Mexico

**Keywords:** Glioma, Mifepristone, Radiotherapy, Chemo-radio-sensitizing

## Abstract

**Background:**

We have investigated the ability of Mifepristone, an anti-progestin and anti-glucocorticoid drug, to modulate the antitumor effect of current standard clinical treatment in glioblastoma xenografts.

**Methods:**

The effect of radiation alone or combined with Mifepristone and Temozolamide was evaluated on tumor growth in glioblastoma xenografts, both in terms of preferentially triggering tumor cell death and inhibiting angiogenesis. Tumor size was measured once a week using a caliper and tumor metabolic-activity was carried out by molecular imaging using a microPET/CT scanner. The effect of Mifepristone on the expression of angiogenic factors after concomitant radio-chemotherapy was determined using a quantitative real-time PCR analysis of VEGF gene expression.

**Results:**

The analysis of the data shows a significant antitumoral effect by the simultaneous administration of radiation-Mifepristone-Temozolamide in comparison with radiation alone or radiation-Temozolamide.

**Conclusion:**

Our results suggest that Mifepristone could improve the efficacy of chemo-radiotherapy in Glioblastoma. The addition of Mifepristone to standard radiation-Temozolamide therapy represents a potential approach as a chemo-radio-sensitizer in treating GBMs, which have very limited treatment options.

## Background

Glioblastoma multiforme (GBM) is the most common tumor of the central nervous system, with a prognosis of 15 months median survival following diagnosis. GBM is a fast-growing glioma that develops from astrocytes, star-shaped glial cells that support nerve cells. GBM is classified as a grade IV astrocytoma, which is the most invasive type of glial tumor. This kind of tumor is highly aggressive, growing rapidly and commonly spreading to nearby brain tissue. Its treatment has been a challenge due to its localization in the brain [1,2].

The standard treatment for GBM is surgery, followed by radiation therapy [3,4] accompanied by chemotherapy. In standard external radiation therapy (radiotherapy), multiple sessions of standard-dose "radiation fractions" are delivered to the tumor site as well as its margin in order to treat the zone of infiltrating tumor cells. Although the goal of radiation therapy is to kill tumor cells selectively while leaving normal brain tissue unharmed, in reality each treatment session induces damage to both healthy and normal tissue, which limits the benefit of radiotherapy.

The radiation dose that can be tolerated by the brain is approximately 60 Gray. However, this dose is inadequate for total tumor eradication, resulting in a poor treatment response in patients with glioma. Consequently, the co-administration of chemotherapy with radiation has been used in recent years with the intention of improving treatment response [5,6].

Chemotherapy with the drug temozolamide is the current standard treatment for GBM. The drug is administered every day during radiation therapy and then in six to eight cycles of five days (with a rest period between each cycle) at higher doses once radiation is completed [7]. While the aim of chemotherapy is long-term tumor control, this goal is reached in only about 20 percent of patients. That is overall patient survival with prolonged remission of GBM tumors has not improved with the addition of chemotherapy to the treatment regimen [8,9]. Frequency of recurrence and rapid tumor progression emphasize the need for treatment alternatives to achieve long-term patient survival. Research efforts currently underway are focused on two approaches: (i) inhibition of angiogenesis in tumors, and (ii) the identification of agents that effectively and preferentially trigger the cell death process in tumors.

The activation of angiogenesis, the formation of new blood vessels from a preexisting vascular network, appears to play an important role in glioma development and progression. Several studies have correlated increased tumor vascularization with impaired patient survival [10,11]. Although there are a variety of factors that promote angiogenesis, the main one is VEGF (vascular endothelial growth factor) a regulator of endothelial cell proliferation and capillary hyperpermeability involved in malignant glioma.

Preclinical reports strongly suggest that antiangiogenic therapy can have a clinical benefit due to a vascular density reduction of tumors leading to a decrease in tumor size [12,13]. However, the current antiangiogenic therapies have shown only a moderate clinical efficacy, with poor results in advanced glioblastoma.

Regarding the other current research focus in this field, one possible agent for selectively triggering GBM tumor cell death is Mifepristone, which is a compound with activity as a progesterone and glucocorticoid receptor antagonist. Consequently, the use of Mifepristone for the inhibition of tumor cell growth has shown positive results principally in hormone-dependent (e.g., breast, prostate and ovarian) cancer.

Recently it was reported that vascular endothelial growth factor (VEGF), epidermal growth factor receptor (EGFR), cyclin D1 and progesterone receptor (PR) expression levels are elevated in patients with high–grade astrocytomas and progesterone regulates astrocytomas growth through its interaction with progesterone receptor [14]. The same authors also demonstrated that progesterone increased VEGF and EGFR expression, and cell proliferation in two human astrocytoma cell lines derived from tumors of different evolution grades (U373 grade III and D54 grade IV) and these effects were inhibited by Mifepristone [14,15]. Other authors have also showed that the expression of VEGF in prostate cancer cells DU 145 and PC3 treated with 10 micromol/L of Mifepristone was significantly decreased [16].

As a chemosensitizing agent, Mifepristone has been used to modulate the cytotoxic activity of doxorubicin [17], paclitaxel [18] and cisplatin [19].

We previously reported that Mifepristone was able to enhance the citotoxicity of cisplatin in cervical cancer cells in vitro and in vivo by increasing the intracellular as intratumoral concentration of cisplatin [20]. In another study using cervical cancer cells [21], we showed that the combination of cisplatin with a pure antiestrogen, ICI 182,780, induced the arrest of the cell cycle at the G2/M phase. The failure of this control checkpoint is believed to lead to genomic instability, resulting in hypersensitivity to radiation. However, the role of Mifepristone, alone or combined with other drugs, has been poorly studied as a chemo-radio-sensitizer.

The aim of the present study was to investigate whether Mifepristone can modulate the growth of glioma xenotrasplants treated with temozolamide and radiation, and/or decrease of VEGF expression.

## Results

**Figure 1:**
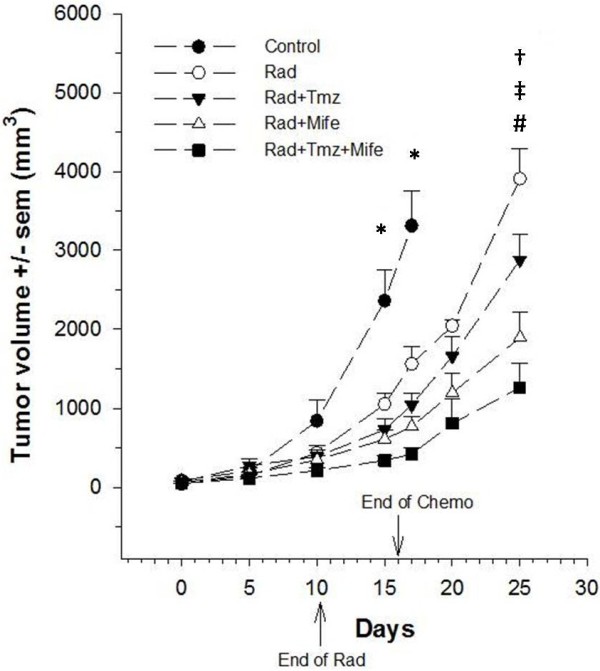
**Antitumor activity on glioblastoma xenografts for control and experimental groups.** Ionizing radiation alone (Rad) or combined with Temozolamide (Rad + Tmz), Mifepristone (Rad + Mife) or both (Rad + Tmz + Mife). Each point represents the average ± SEM of four to five animals. (*) indicates a significant difference (p < 0.05) between the control and experimental groups; (†) and (‡) represent a significant difference between Rad + Tmz + Mife vs. Rad and Rad + Tmz + Mife vs. Rad + Tmz, respectively. (#) indicates a significant difference between Rad vs. Rad + Mife.

After treatment of glioma xenografts with chemo-radiotherapy, tumor growth was evaluated (Figure 1). The tumor volume from control animals is showed up to day 17. Due to the large size of tumors, animals were sacrificed on that day. The statistical analysis shows no differences in tumor growth during the first 10 days, but starting from day 15 there was indeed a significant decrease in this value (*p* < 0.05) between the control and all experimental groups. At day 17 the reduction in tumor volume fluctuated between 2.5-fold, (Control vs. Irradiation alone: Rad) and 7-fold (Control vs. the combination of both chemotherapeutic agents and irradiation: Rad + Tmz + Mife). At day 25, the statistical analysis indicates that tumor volume of Rad + Tmz + Mife group was significantly less from that of the Rad group (Power = 0.84) and Temozolamide group (Rad + Tmz) (Power = 0.84), but not from than of the Mifepristone group (Rad + Mife). Statistical analysis also indicates a significantly lesser tumor growth in the Rad + Mife group than the Rad group (Power = 0.95). However, no difference was found in tumor growth between Rad vs. Rad + Tmz, or between Rad + Tmz vs. Rad + Mife.

**Figure 2:**
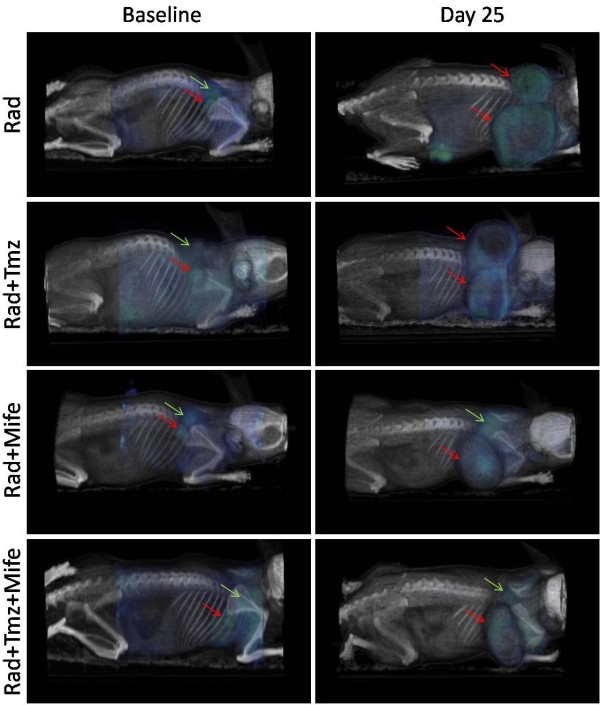
**PET/CT images showing **^**18**^**F-FDG tumor uptake.** The groups are: Rad, Rad + Tmz, Rad + Mife and Rad + Tmz + Mife. Left images represent the beginning of treatment (baseline) and right images the end of the study (day 25). Red arrows indicate tumor location at baseline and day 25; green arrows show sites of typical ^18^F-FDG uptake in brown adipose tissue (BAT).

Figure 2 shows tumor PET/CT image of a representative animal from each of the Rad, Rad + Tmz, Rad + Mife and Rad + Tmz + Mife groups. Left images are baseline at the beginning of treatments, and right images are at day 25. Blue indicates ^18^F-FDG uptake, red arrows indicate tumor location at baseline and day 25; green arrows show sites of typical ^18^F-FDG uptake in brown adipose tissue (BAT) in the neck of the mice. The physiological ^18^F-FDG uptake in areas of supraclavicular fat has recently been recognized as ^18^F-FDG uptake in brown adipose tissue (BAT) using PET/CT technology. BAT functions as a thermogenic organ by producing heat to maintain body temperature in mammals, and it is generally in deep cervical regions including the supraclavicular areas. BAT is known to exhibit increases glucose uptake when the sympathetic nervous system is activated by cold stimulation [22]. Accordingly, the anesthesia used in the animals subjected to PET/CT assays can decrease the temperature slightly at the moment of imaging. In our study, due to fact that the anesthesia used appeared to decrease the temperature at the moment of imaging, we observed a typical ^18^F-FDG uptake in BAT in the neck of animals.

**Figure 3:**
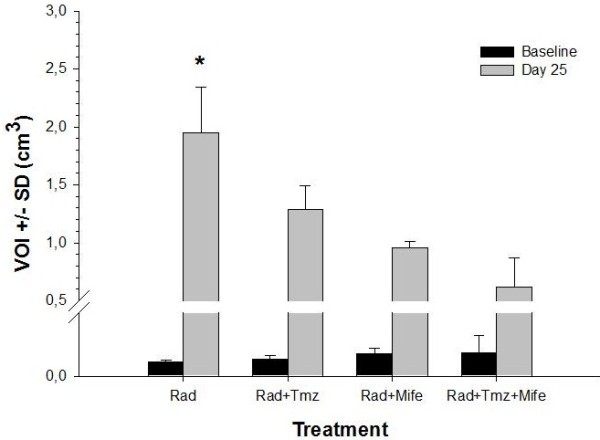
**Metabolic activity of tumors, in terms of the VOI for the different experimental groups.** (*) indicates a significant difference between the Rad group and the other experimental groups (n = 2).

Images show the changes in metabolic activity after treatments. Metabolic tumor volume, represented in terms of the VOI [23], is depicted in Figure 3. There was a significant difference between the VOI from the Rad group and that of the groups with combined chemo-radiotherapy treatments.

**Figure 4:**
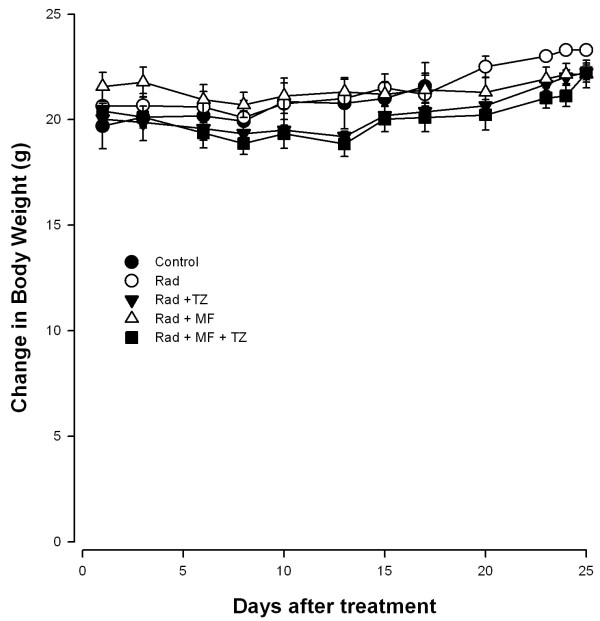
**Evaluation of body weight change.** Mice treated with radiation alone (○), radiation combined with Mifepristone (Δ) or Temozolamide (▼), or radiation with the combination of both (■). Controls (●) were treated only with vehicle. There was no significant difference between groups. Data are presented as the means ± SEM of four or five animals.

The toxicity of treatments is shown in the Figure 4. No change in weight was observed, indicating no systemic toxicity with any of the treatments (radiotherapy alone or combined with chemotherapy). Although there was light weight loss at the beginning of the combination treatment with irradiation plus Mifepristone and Temozolamide, the weight of the animals in this group returned to the pretreatment values by the end of the study.

**Figure 5:**
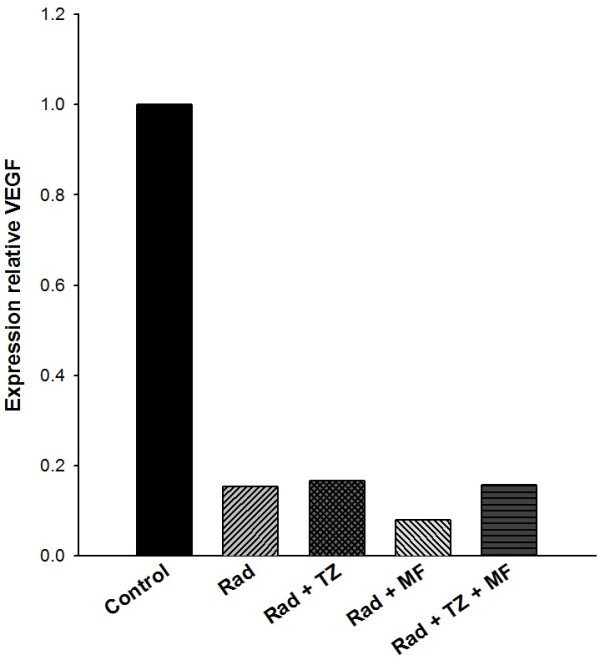
**Quantitative Real-Time PCR analysis of VEGF relative expression in C6 xenografts after each treatment.** Analysis was done on whole lysates from tumors removed on day 25.

Figure 5 shows the analysis of VEGF expression levels in tumors at the end of the study. The results indicate that compared to the control tumors, there was a significant decrease in this value after all treatments (radiotherapy alone or radiotherapy combined with Mifepristone and/or Temozolamide). No difference was observed between any of the groups with treatments, demonstrating that ionizing radiation alone or its combination with any of the tested chemotherapy agents caused a marked reduction in VEGF expression.

## Discussion

To date, there is no effective therapy for GBM, a highly aggressive tumor whose treatment has been a permanent challenge. The current treatments of high-grade glioma patients with the combination of radiation and chemotherapy can result in substantial lymphopenia, immunosuppression and opportunistic infections compromising patient survival [24]. Therefore, new alternatives are needed in order to increase patient survival and avoid recurrence.

The present study aimed to evaluate whether the introduction of Mifepristone, a progesterone and glucocorticoid receptor antagonist, to standard therapy with Temozolamide and radiation improves the response of glioma tumor *in-vivo*. The role of this antihormonal agent as a chemo-radio-sensitizer in GBM treatment has scarcely been explored.

We observed significant differences in tumor volume growth between the control and treated groups (Figure 1). Moreover, when Mifepristone was administered concomitant with radiation, tumor growth rate was slower than with the radiation alone. Furthermore, the reduction of tumor growth-rate was even more evident when we added Mifepristone to the Temozolamide-Radiation scheme. This suggests that Mifepristone can play an important role in the chemo-radio-sensitization of GBM xenografts.

PET/CT images (Figure 2) illustrate the effect of the different treatments, showing not only differences in tumor size at day 25 but also variations in ^18^F-FDG uptake in tumors, denoted by the blue-green color in the tumor mass. The later is better visualized in terms of the VOI values measured in the metabolic images from PET (Figure 3); as mentioned before, the VOI measures the volume concentration of ^18^F-FDG in the tumor and represents its metabolic activity. In this experiment we observed that the size of the VOI (in cm^3^ or mm^3^) is not always equal to the anatomical volume of the tumor (compare ordinate-scale in Figures 2 and 3), as result of the existent necrosis in some tumors (gray regions in tumors from Rad and Rad-Tmz groups at day 25 in Figure 2) where no uptake is observed. For this reasons the VOI should be used as a more representative way to evaluate the therapeutic response in tumors under treatment. Our group is working in this topic by performing some comparative studies between the simple measurement of the dimension of the tumor and the VOI.

The Mifepristone dose used in our study was chosen in accordance with previous reports in which a tumor growth rate inhibition in ovarian and prostate xenografts was observed [25,26]. However, the total accumulated dose used in the present study was lower compared to that used in any of the reports in the literature. Interestingly, when patients with non-resectable meningioma were chronically exposed to Mifepristone, no severe side effects were observed [27]. Thus Mifepristone can be administered for prolonged periods. In addition our results demonstrated no significant change in the body weight of animals, suggesting that Mifepristone could be administered at higher doses.

Previous studies have shown that Mifepristone induces G1-S blockage of the cell cycle through inhibition of cdk2 activity in human ovarian cancer cells [25]. A reduction in cdK2 activity has been associated with an inhibition of the transcription factor E2F1, which modulates S-phase progression [28,29]. Accordingly, the induced chemo-radiation-damage found in the present study might owe itself to the capacity of Mifepristone to arrest the G1-S Phase of the cell cycle.

Another possible mechanism involved in the sensitizer effect of this antihormonal agent is its antagonist action on progesterone and glucocorticoid receptors. It is widely accepted that progesterone participates in the development of different types of cancer as a transcription factor [14,30,31]. It was reported [14,15] that progesterone-receptor isoforms are expressed in two cell lines (U373 and D54) derived from high-grade human astrocytoma. The same authors also demonstrated that progesterone increased VEGF and EGFR expression and cell proliferation, Mifepristone was able to inhibit not only the progesterone effects but also when it was administered alone significantly reduce astrocytoma cell growth in vitro.

It was reported that Mifepristone binds strongly to glucocorticoid receptors, being its binding affinity for these receptors approximately five and three times greater than progesterone and dexamethasone, respectively. Since the glioma C6 cells used in our study have an elevated expression of glucocorticoid receptors, the blockage of these receptors by Mifepristone probably led to the receptor transactivation inhibition and therefore the inhibition of cell proliferation.

Considering the extensive evidence that glioma cells produce high levels of VEGF, and Mifepristone decreases the expression of VEGF in prostate cancer cells [16], breast cancer [32] and gastric cancer cells [33]; we also investigated the possible participation of Mifepristone in the inhibition of VEGF expression in glioma xenografts.

It was reported that the use of antiangiogenic therapies in high-grade gliomas results in VEGF inhibition, improving the vascular function and tumor oxygenation that can increase the response to radiation [34].

To elucidate whether the treatment with radiation-Mifepristone-Temozolamide acted on angiogenesis, we evaluated the VEGF expression in GBM xenografts at end of the experiments. However, radiation alone was enough to drastically decrease VEGF production, so in our experimental conditions was not possible to prove the direct participation of Mifepristone on VEGF down-regulation (Figure 5). Therefore, in future studies will be necessary to performed different schemas of the treatments that demonstrate the influence of Mifepristone on VEGF expression.

More studies should be performed to understand the mechanism by which Mifepristone acts, either by itself or in combination with radiation and other drugs, to inhibit tumor growth. Tieszen CR et al. [35] reported that growth inhibition of cancer cells by antiprogestin Mifepristone is not dependent upon expression of nuclear progesterone receptors. They showed that Mifepristone is able to inhibit the growth of *in vitro* cancer cells derived from the nervous system, breast, prostate, ovary, and bone, with an absence of expression of classic nuclear progesterone receptor in nearly all these cells.

Consequently, the potential action of Mifepristone in chemo-radiation treatments of different tumors may be mediated by other mechanisms, including its participation in apoptosis, cell cycle arrest, and expression of ATM or other radiosensitizer proteins, mechanisms that have been observed in other cell types and therefore may also be contributing to the reduction in size of glioblastoma xenografts found presently (Figure 1).

## Conclusion

The present study suggests several possible mechanisms for the significant decrease in GBM tumor size found with the addition of Mifepristone to the treatment with radiation or radiation plus temozolamide. Whatever the possible mechanism, the current results strongly suggest the potential of Mifepristone as a chemo-radio-sensitizer for the standard treatments of GBM tumors, for which currently available treatments have shown limited effects.

Future studies are necessary to explain the mechanisms related to the chemo-radio-sensitizing effect of Mifepristone in GBM, not only on tumor xenografts but also in ortotopic models of glioma.

## Methods

### Drugs and reagents

Mifepristone, Temozolamide and Trypsin were obtained from Sigma Chemical Co. (St. Louis, MO, USA). Dulbecco´s modified Eagle’s medium (DMEM), FCS (fetal calf serum), EDTA (Ethylenediaminetetracetic acid), Tris and SDS were obtained from Gibco, BRL (Grand Island, NY, USA). High-quality water employed to prepare solutions was obtained through a Milli-Q Reagent Water System (Continental Water Systems; El Paso, TX, USA).

### Solutions

A stock solution (1 mg/mL) of Temozolamide was prepared in DMSO, and Mifepristone was reconstituted in Polietilenglicol-saline solution in a 50:50 mixture. All standard solutions were stored at −20°C until use.

### Animals

Female athymic Balb-C nu/nu mice, between 6–8 weeks of age, were supplied by the Instituto Nacional de Nutrición (INCMNSZ), Mexico City, Mexico. All animals were kept in a pathogen-free environment and fed *ad lib.* The procedures for care and use of the animals were approved by the Ethics Committee of the Instituto Nacional de Cancerología (INCan) (Mexico City, Mexico), and all applicable institutional and governmental regulations concerning the ethical use of animals were followed.

### Cell cultures

The glioma C6 cell line used in this study (obtained from ATCC® CCL-107™, Rockville, Maryland, USA) was cloned from a rat glial tumor induced by N-nitrosomethylurea by Benda et al. [36]. This cell line was routinely maintained as a monolayer in DMEM supplemented with 5% fetal bovine serum and incubated at 37°C in a 5% CO_2_ atmosphere at high humidity. Cells were harvested with 0.025% Trypsin and 1 mM EDTA.

### Tumor xenografts

Mice were subcutaneously (s.c.) inoculated with 1x10^6^ C6-cells in the right flank. After inoculation, weekly measurements of tumors were made. Two perpendicular diameters were measured by using a caliper, and tumor volume was determined by using the following relation: V = π/6 × (large diameter × [short diameter]^2^). Once tumors had reached approximately 50 mm^3^, the animals were pair-matched into treatment and control groups and the treatments were initiated. Each group consisted of 4–5 tumor-bearing mice.

### Irradiation procedure

Animals were anaesthetized with 1–3% isoflurane in 100% oxygen by using an animal anesthesia inhalation unit (Bickford, Wales Center, NY), and irradiated with an orthovoltage X-ray unit (D3225, Gulmay Medical Ltd.,UK), as described previously [37]. Animals received fractionated doses of 1 Gy per day for 10 days (Monday to Friday for two weeks). The dose and the schedule were selected in according to a dose–response curve constructed in a previous pilot study. This curve showed a 10 Gy dose as the ED_50_ (Dose of radiation to achieve 50% tumor growth inhibition). The X-ray beam was centered on the tumor lobe by using one of the different lead collimators [37], depending on the tumor size at the moment of irradiation.

### Chemo-radiotherapy

Animals selected for this study were arranged in five groups (n = 4–5 each), including: A) radiation treatment alone (1 Gy/day for 10 days); B) irradiation (the same scheme as in A) combined with Temozolamide (10 mg/kg/day, i.p.); C) irradiation combined with Mifepristone (12.5 mg/kg/day, s.c.); and D) irradiation combined with Mifepristone and Temozolamide (the same scheme as in B and C). Mifepristone and Temozolamide were administered in three cycles during three weeks, each cycle consisting of three consecutive days (from Monday to Wednesday). Control animals received only the vehicle no irradiation. After each drug administration, mice were weighed and the tumor volume was calculated, as previously described (every five days). The experiment was conducted during twenty-five days, at the end of which time all animals were weighed and euthanized.

### Tumor metabolic-evaluation by molecular imaging

Assessment of the metabolic tumor response was performed in two animals of each group using a microPET/CT scanner (Albira ARS, Oncovision Spain). PET/CT images were acquired at the beginning of each treatment, and before euthanization. Mice were imaged under isoflurane anesthesia (1–3% isoflurane in 100% oxygen), 30 min. after the intravenous injection of 200 μCi of ^18^F-FDG (fluorodeoxiglucose) by the caudal vein. Metabolic tumor response, evaluated as ^18^F-FDG uptake by active tumor cells, was measured by means of the VOI (Volume of Interest). The latter is a tool that determines the volume concentration of the radiopharmaceutical (volume radioactivity) in the entire tumor, and represents the metabolic activity of the tumor [23]. VOIs were measured over the tumor images by using the image analysis software PMOD (PMOD Technologies Ltd.) from regions with ^18^F-FDG uptake in tumors. Average VOI-values in each group were compared to depict tumor metabolic differences resulting from the different treatments.

### VEGF expression analysis with Quantitative Real-Time PCR

The effect of Mifepristone on the expression of angiogenic factors during concomitant chemo-radiotherapy was examined using quantitative real-time PCR (QrtPCR). VEGF expression levels in the tumor tissue from glioma xenografts were evaluated at the end of the study. Briefly, the whole tumors were lysed and the total RNA was isolated from each tumor with a method based on guanidine isothiocyanate/phenol/chloroform extraction using TRIzol reagent (Invitrogen Life Technologies) and quantified with UV spectroscopy. After quantification, 200 ng of total RNA was used in the presence of the TaqMan® RNA-to-CT™ 1-Step Kit (Applied Biosystems) to perform one-step RT-PCR TaqMan Gene Expression Assays of vascular endothelial growth factor A (VEGF-A) (Hs00900055_m1, Applied Biosystems) by using a FAM probe and Endogenous Control Human GAPDH (4310884E, Applied Biosystems) with VIC Probe. Real-time quantization was realized on the Spectrum 48 thermocycler Instrument (Esco, Micro Pte Ltd, Singapore).

PCR reactions were carried out in a total volume of 10 μL. The reaction conditions were as follows: pre-incubation at 60°C for 15 min and 94°C for 5 min, followed by 40 cycles (amplification) at 94°C for 15 s and 60°C for 60 s. Fluorescence emission spectra were monitored and analyzed. PCR products were measured by the threshold cycles (TC), at which specific fluorescence became detectable. The TC was used for kinetic analysis and was proportional to the initial number of target copies in the sample. Analysis of relative gene expression was based on the 2^-ΔΔCT^method. The analysis was carried out with four to five independent samples.

### Statistical analysis

Values are reported as the mean ± SEM. Statistical analysis was performed using one-way analysis of variance (ANOVA) to compare tumor volumes or VOIs between groups, using SPSS Base 20.0 software (SPSS Inc., Chicago, IL). Differences were statistically compared using multiple comparisons between-groups. When necessary, comparison of means was Bonferroni adjusted. A log transformation was applied to the data to better satisfy the assumptions underlying the analysis. The means and standard errors were computed from untransformed data and analysis of statistical significance (p < 0.05) was based on transformed data. A statistical power of analysis was done and a (1-β) > 0.80 was considered sufficient to reject the possibility of a Type I error [38].

## Abbreviations

GBM: Glioblastoma multiforme; DMEM: Dulbecco´s modified Eagle’s medium; FCS: Fetal calf serum; EDTA: Ethylenediaminetetracetic acid; VOI: Volume of Interest; RT-PCR: Reverse-transcriptase polymerase chain reaction; cdk2: Cyclin-dependent kinase 2; BAT: Brown adipose tissue.

## Competing interests

The authors declare that they have no conflict of interests with any products mentioned in the study.

## Authors’ contributions

PGL and LAM planned the studies, coordinated all activities, performed analysis of results, and were involved in the experimental procedures. MLM participated in the experimental procedures of chemo-radiotherapy, molecular imaging acquisition and data processing. RJ participated in the tumor model design, chemotherapy procedures and VEGF expression experiments. MRP participated in the molecular imaging experiments and image analysis. All authors read and approved the final manuscript.
